# Epstein-Barr Virus Encoded dUTPase Containing Exosomes Modulate Innate and Adaptive Immune Responses in Human Dendritic Cells and Peripheral Blood Mononuclear Cells

**DOI:** 10.1371/journal.pone.0069827

**Published:** 2013-07-22

**Authors:** Maria Eugenia Ariza, Pierre Rivailler, Ronald Glaser, Min Chen, Marshall V. Williams

**Affiliations:** 1 Department of Molecular Virology, Immunology, and Medical Genetics, The Ohio State University College of Medicine, Columbus, Ohio, United States of America; 2 Institute for Behavioral Medicine Research, The Ohio State University College of Medicine, Columbus, Ohio, United States of America; 3 Department of Biological Sciences, University of South Carolina, Columbia, South Carolina, United States of America; 4 Comprehensive Cancer Center, The Ohio State University, Columbus, Ohio, United States of America; University of Nebraska-Lincoln, United States of America

## Abstract

We have recently demonstrated that Epstein-Barr virus (EBV)-encoded deoxyuridine triphosphate nucleotidohydrolase (dUTPase) modulates innate immunity in human primary monocyte-derived macrophages through toll-like receptor (TLR) 2 leading to NF-κB activation and the production of pro-inflammatory cytokines. Our previous depletion studies indicated that dendritic cells (DCs) may also be a target of the EBV-encoded dUTPase. However, the role of EBV-encoded dUTPase in DC activation/function and its potential contribution to the inflammatory cellular milieu characteristic of EBV-associated diseases remains poorly understood. In the present study, we demonstrate that EBV-encoded dUTPase significantly altered the expression of genes involved in oncogenesis, inflammation and viral defense mechanisms in human primary DCs by microarray analysis. Proteome array studies revealed that EBV-encoded dUTPase modulates DC immune responses by inducing the secretion of pro-inflammatory T_H_1/T_H_17 cytokines. More importantly, we demonstrate that EBV-encoded dUTPase is secreted in exosomes from chemically induced Raji cells at sufficient levels to induce NF-κB activation and cytokine secretion in primary DCs and peripheral blood mononuclear cells (PBMCs). Interestingly, the production of pro-inflammatory cytokines in DCs and PBMCs was TLR2-dependent. Together these findings suggest that the EBV-encoded dUTPase may act as an intercellular signaling molecule capable of modulating the cellular microenvironment and thus, it may be important in the pathophysiology of EBV related diseases.

## Introduction

Epstein-Barr virus (EBV) is a gamma herpesvirus that is implicated in the pathogenesis of a variety of human malignancies including Burkitt’s lymphoma (BL), nasopharyngeal carcinoma (NPC), Hodgkin’s disease (HD), chronic lymphocytic leukemia (CLL), diffuse large B-cell lymphoma, NK/T-cell lymphoma, and gastric carcinoma [1]. EBV infects a significant percentage (>90%) of the worldwide population and establishes a life-long persistent infection in memory B-cells. However, the various strategies that EBV employs to prevent its clearance and that allow for the establishment and maintenance of a persistent infection in immunocompetent individuals are poorly understood.

Dendritic cells (DCs) are professional antigen presenting cells (APC) and play a pivotal role in regulating the balance between immunological tolerance and immune responses that initiate innate and adaptive immunity. Given the importance of DCs in initiating an immune response against pathogens and the ability of EBV to establish persistent infections in the host, it might be expected that the virus has developed mechanism(s) to regulate the function of DCs as part of the virus strategy to evade immune surveillance. However, the interactions between DCs and EBV remain unclear and conflicting results have been reported concerning the ability of EBV to induce productive infections in DCs. Li et al [2] and Wang et al [3] reported that infection of monocytes by EBV results in apoptosis, thus, preventing their differentiation into DCs. Conversely, Walling et al [4] demonstrated that EBV established a latent infection in blood-borne mononuclear cells, which are precursors of Langerhans cells (LC) and that upon migration and differentiation into LC in the epithelium, EBV is reactivated establishing a productive (lytic) infection. These conflicting results suggest that while EBV may be able to infect monocytes, the effect of EBV on these cells varies with some precursors undergoing apoptosis, while infection of other precursors leads to the establishment of either a latent or productive infection.

The EBV-encoded deoxyuridine triphosphate nucleotidohydrolase (dUTPase), which was identified by our laboratory [5], is encoded by the BLLF3 gene and expressed as an early protein during lytic replication [5], [6]. The EBV-encoded dUTPase protein has been detected using immunohistochemical techniques in the upper epithelial layers of oral hairy leukoplakia (HL) lesions, in lymphoid cells from tonsils of patients with infectious mononucleosis (IM) and in NPC tissue [7], [8]. Expression of BLLF3 has also been detected in EBV genome positive tumor cell lines established from patients with nasal NK/T-cell lymphoma using microarray technology [9]. Our recent studies have demonstrated that the EBV-encoded dUTPase possesses novel functions in innate/adaptive immunity, independent of its enzymatic activity, due in part to the stimulation of toll-like receptor (TLR) 2 and subsequent activation of NF-κB leading to the induction/secretion of pro-inflammatory cytokines [10]–[12]. These studies indicated that the primary cellular targets of the EBV-encoded dUTPase were monocytes/macrophages and DCs. To gain insight into the biological effects of EBV-encoded dUTPase on human DCs (hDCs) function we evaluated gene expression changes by microarray analysis following treatment of hDCs with the EBV-encoded dUTPase and compared it to that of untreated cells using U133 Plus 2.0 Human genome GeneChips. The results from this study revealed that EBV-encoded dUTPase significantly altered the expression of genes involved in oncogenesis, inflammation and viral defense mechanisms. Additional proteome array studies of hDC supernatants following treatment with the EBV-encoded dUTPase demonstrated that the EBV-encoded dUTPase modulated DC function by activating cytokine/chemokine-receptor signaling pathways leading to the production of T_H_1 and T_H_17 pro-inflammatory cytokines. Finally, we demonstrate that the EBV-encoded dUTPase is secreted in exosomes of chemically induced Raji cells and that these exosomes induced the secretion of cytokines and chemokines through TLR2 in human primary cells. These results suggest that the EBV-encoded dUTPase by being secreted from cells undergoing lytic and/or abortive-lytic replication can modulate immune functions of neighboring cells and thus, alter the microenvironment to contribute to the pathophysiology of EBV-associated diseases.

## Materials and Methods

### Reagents

The NF-κB luciferase promoter construct pNF-κB-Luc and the transfection control reporter vector pRL-TK, were purchased from Clontech Laboratories, Inc., (Mountain View, CA), and Promega (Madison, WI), respectively. Blasticidin and Pam3CSK4 were purchased from Invivogen (San Diego, California). IgG2a Isotype control monoclonal antibody was purchased from eBioscience and anti-TLR2 (clone TL2.1) monoclonal antibody was purchased from Imgenex (San Diego, California).

### Purification of the EBV-encoded dUTPase

Detailed methods for the purification of EBV-encoded dUTPase have been previously reported by our group [10]–[12]. All EBV-encoded dUTPase preparations were tested as described previously [10] and were free of detectable levels of LPS, peptidoglycan (SLP-HS), DNA or RNA. Protein concentration was determined with a Coomassie Brilliant Blue dye-binding assay (Bio-Rad Laboratories, Hercules, CA) using bovine serum albumin as the standard. The purified EBV-encoded dUTPase used in these studies was stored at−80°C at stock concentrations of 0.2 and 0.5 mg/ml.

### Cell Culture

Human dendritic cells (hDC/LCs; myeloid, plasmacytoid and Langerhan cells) were obtained from MatTek Corporation (Ashland MD). These cells were generated from CD34+ progenitor cells derived from human umbilical cord blood (HUCB) cells and cultured using specially formulated medium, DC-100-MM, (MatTek) containing a cytokine cocktail designed to induce differentiation of the CD34^+^ into DCs. These DCs express surface markers CD1a, HLA-DR, co-stimulatory molecules, Birbeck granules and surface markers characteristic of both plasmacytoid and myeloid DC [13].

Human embryonic kidney 293 (HEK293) cells stably expressing human TLR2 were purchased from Invivogen (San Diego, CA). Cells were maintained in DMEM supplemented with L-glutamine (2 mM), HEPES (10 mM), sodium pyruvate (1%), and 10% heat-inactivated FBS, plus 10 µg/ml blasticidin (HEK293-TLR2).

Raji cells, an EBV-genome positive Burkitt’s lymphoma nonproducing cell line, were maintained in RPMI-1640 supplemented with L-glutamine (2 mM), HEPES (10 mM), sodium pyruvate (1%), and 10% heat-inactivated FBS at 37°C, 5% CO_2_ and 95% humidity. For exosome production, Raji cells were seeded at 1×10^6^ cells/ ml and treated with 12-0-tetradecanoylphorbol-13 acetate (TPA; 40 ng) and 3 mM sodium butyrate for 72 h to induce replication of EBV or left untreated. Following treatment, culture supernatants were collected for exosome isolation.

Human peripheral blood mononuclear cells (PBMCs) from healthy subjects were obtained from Astarte Biologics (Cat# 1001 Lot # 1704OC12).

### Gene Expression Analysis in Hdcs

The effect of the EBV-encoded dUTPase on gene expression in hDCs was determined by treating hDCs with purified EBV-encoded dUTPase (10 µg/ml), as we have described [12], for 4 h and then comparing the gene expression in these cells to untreated controls. A total of 5 samples (2 untreated controls and 3 EBV-encoded dUTPase treated) were employed in this study. After treatment, total RNA was isolated using the RNAqueous-4PCR kit (Ambion, Grand Island, NY), following the procedure supplied by the manufacturer. Trace amounts of DNA were removed by treatment with DNase 1 and the RNA quantity and integrity was analyzed using a 2100 Bioanalyzer (Agilent, Santa Clara, CA). For gene expression studies, sample preparation and processing procedures were performed as described in detail in the Affymetrix GeneChip Expression Analysis Manual (Santa Clara, CA). Briefly, cRNA was synthesized from 3.5 µg of total RNA using a one-cycle reaction followed by biotin labeling of antisense cRNA and then hybridized to human genome U133 Plus 2.0 GeneChips (Affymetrix Santa Clara, CA). The GeneChips were washed using the automated Affymetrix fluidics station and the bound biotin-labeled cRNA detected using a Streptavidin-Phycoerythrin conjugate. Subsequent signal amplification was performed using a biotinylated anti-streptavidin antibody followed by Genechip scanning. All microarray data have been deposited in NCBI’s Gene Expression Omnibus and are accessible through GEO Series accession number GSE46519 (http://www.ncbi.nlm.nih.gov/geo/query/acc.cgi?acc=GSE46519).

Affymetrix GeneChip hybridization data were normalized using two methods: Robust Multi-Array expression using sequence information (GCRMA)^−^ [14] and Model-Based Expression Indexes (MBEI) [15]. GCRMA normalization was performed using the online analysis tool ArrayQuest^−^ [16] to implement the Bioconductor GCRMA package (http://www.bioconductor.org/). MBEI normalization was performed using the dChip analysis tool. Differentially expressed genes between control and treated samples were then identified for each normalized data set using the criteria fold change >2 and *p*<0.05 (un-paired t-test on the comparison between the expression values of control and treated samples). Genes overlapping between the GCRMA and MBEI gene lists were used for further analysis. GO and KEGG annotations were retrieved for these genes using the Affymetrix NetAffx analysis tool and gene expression patterns were clustered using dChip unsupervised clustering algorithm with centroid linkage [17].

### Quantitative Real-time PCR

The expression of a select group of differentially expressed genes identified by microarray analysis was validated using pre-designed and functionally validated TaqMan assays on demand (Applied Biosystems, Grand Island NY). RNA from untreated or EBV-encoded dUTPase treated hDC/LCs for 4 h was examined by qRT-PCR for the expression of CCL20, IL-6, CXCL11, IFIT1, IFIT2, IFIT3, OASL, OAS2, TNFAIP6 and IF44, FOXO1 using ABI specific TaqMan gene expression assays/ABI primer reference (Hs00171125_m1, Hs00174131_m1, Hs00171138_m1, Hs00533665_m1, Hs00155468_m1, Hs01911452_m1, Hs00984390_m1, Hs00942650_m1, Hs01113602_m1, Hs00951344_m1, and Hs01054576_m1). Samples were normalized to GAPDH (Hs99999905_m1) and expressed as the mRNA expression levels relative to untreated control. All reactions were performed in triplicate on an ABI 7900HT System.

### Western Blot Analysis

The expression of a select group of differentially expressed genes identified by microarray analysis was also validated by western blot. Whole cell lysates (25 µg) from untreated or EBV-encoded dUTPase-treated hDC/LCs for 4 h were size-fractionated in a polyacrylamide gel under standard SDS-PAGE conditions. Proteins were then transferred onto PVDF membranes (Bio-Rad Laboratories, Hercules, CA) and analyzed by immunoblotting using anti-CCL20 (1∶200 dilution), anti-CXCL11 (1∶200 dilution), anti-IFIT3 (1∶200 dilution) (Santa Cruz Biotechnology, Santa Cruz, CA) or anti-β-actin antibody (1∶15,000; Sigma Chemical Co, St. Louis, MO). HRP-conjugated secondary antibodies were used at 1∶4000 and the signal detected by enhanced chemiluminescence.

Detection of exosomal marker proteins was determined by western blotting using the exosome antibody kit, as described by the manufacturer (SBI System Biosciences Mountain View, CA). Briefly, exosome extracts (25 µg) from non-induced or TPA and sodium butyrate induced Raji cells for 72 h, were separated in a polyacrylamide gel under standard SDS-PAGE conditions. Proteins were then transferred onto PVDF membranes and analyzed by immunoblotting as described above using anti-HSP70 (1∶1000 dilution) and HRP-conjugated secondary goat anti-rabbit antibody (1∶20,000 dilution), as recommended by the manufacturer.

### Luciferase Reporter Gene Assays

TLR2-HEK293 expressing cells (2.5×10^5^) were seeded into 12-well plates and transiently transfected 24 h later using lipofectamine 2000 transfection reagent (Invitrogen; Carlsbad, CA), as we have previously described [12], [18]. Briefly, cells were transfected with pNFκB-Luc (0.5 µg) and pRL-TK (8 ng) reporter vectors. At 24–36 h following transfection, cells were treated with intact exosomes or exosomes extracts from non-induced or TPA and sodium butyrate induced Raji cells for 72 h, culture supernatants (30 ml) from non-induced or chemically induced cells concentrated 10-fold using Centricon PL-10, or purified EBV-encoded dUTPase (10 µg/ml) for 8 h or left untreated. Following treatment, cell lysates were prepared and reporter gene activities were measured using the dual-luciferase reporter system (Promega, Madison, WI). Data was normalized for transfection efficiency and reporter activity expressed as the mean relative stimulation ± SD.

### Cytokine/Chemokine Proteome Array

Human primary dendritic cells (3×10^5^), were treated with EBV-encoded dUTPase protein (10 µg/ml) for 24 h and the levels of twelve cytokines, chemokines in cell culture supernatants of treated and control samples were measured using SearchLight**®** proteome arrays (Aushon BioSystems, Billerica, MA), as we have described [18]. Briefly, samples were incubated for 1 h on the array plates that were pre-spotted with capture antibodies specific for each protein biomarker. The bound proteins were detected with a biotinylated detection antibody, followed by the addition of streptavidin-horseradish peroxidase (HRP) and lastly, a chemiluminescent substrate. The plates were immediately imaged using the SearchLight**®** imaging system, and data was analyzed using SearchLight**®** Array Analyst software. The amount of luminescent signal produced is proportional to the amount of each protein present in the original standard or sample. Concentrations are expressed as pg/ml and represent the average ± SD of an n of 4.

### Purification of Exosomes

Raji cells were induced with TPA and sodium butyrate for 72 h. Induced or non-induced Raji cell cultures were centrifuged at 3000×g for 15′ to remove cells and cell debris and the supernatants were transferred to a sterile tube. Culture supernatants (30 ml) were either concentrated 10-fold using Centricon PL-10 or used for exosome purification using the ExoQuick-TC™ precipitation method. This method was employed based on literature reports [19] demonstrating that ExoQuick precipitation produces exosomal RNA and protein with greater purity and quantity than chromatography, ultracentrifugation and DynaBeads. Briefly, ExoQuick-TC™ precipitation solution (2 ml; SBI System Biosciences Mountain View, CA) was added to the supernatant (10 ml) from induced and non-induced cells, incubated at 4°C for 48 h and the exosomes were collected by centrifugation as described by the manufacturer. Exosomes were resuspended in PBS and either lysed by freezing and thawing three times or left intact and subsequently used for cell stimulation studies. Additional exosomes preparations were lysedusing a general lysis buffer (50 mM Tris-HCl, pH 7.5 containing 150 mM NaCl and 1% Nonidet-40) and used for dUTPase enzyme assays.

### dUTPase Activity in Exosomes

The dUTPase activity present in exosome lysate preparations and/or in culture supernatants from induced and non-induced Raji cells, obtained as described above, was determined using the standard assay [10] in the presence and absence of human serum from a patient with EBV genome positive diffuse large B-cell lymphoma, which we previously demonstrated contained specific neutralizing antibodies against the EBV-encoded dUTPase.

### Cytokine Profile Induced By Exosomes

For cytokines experiments with exosomes, dendritic cells and PBMCs were seeded at a density of 3×10^5^ in 24-well plates and cultured in AIM-V serum-free medium supplemented with L-glutamine (2 mM), streptomycin (50 µg/ml) and gentamycin (10 µg/ml). The next day, cells were treated with either intact exosomes or exosomal extracts from non-induced or chemically induced Raji cells, EBV-encoded dUTPase (0.1 µg/ml or 10 µg/ml), Pam3csk4 (0.1 µg/ml) or left untreated for 24 h. Following treatment, cell supernatants were collected and the levels of 7 cytokines in treated and control samples were measured by ELISA (MSD Multi-array and Multi-spot human cytokine kit) using a Sector Imager 2400 instrument and the MSD Data Analysis Toolbox software. Concentrations are expressed as pg/ml and represent the average ± SD of an n of 4.

### hTLR2 Blocking Experiments

For blocking experiments, hDCs and PBMCs were seeded at a density of 3×10^5^ in 24-well plates and cultured in AIM-V serum-free medium supplemented with L-glutamine (2 mM), streptomycin (50 µg/ml) and gentamycin (10 µg/ml). The next day, cells were pretreated with (10 µg/ml) anti-human TLR2 monoclonal antibody (anti-TLR2 MAb; IgG2a, clone TL2.1) or IgG2a MAb isotype control for 1 h at 37°C and subsequently exposed to either intact exosomes or exosomal extracts from non-induced or chemically induced Raji cells, Pam3csk4 (0.1 µg/ml) or left untreated for 24 h. Following treatment, cell supernatants were collected and the levels of TNF-α in treated and control samples were measured using the MSD human cytokine assays kit, a Sector Imager 2400 instrument and the MSD Data Analysis Toolbox software. Concentrations are expressed as pg/ml and represent the average ± SD of an n of 4.

### Statistical Analysis

The statistical comparisons between different study groups were carried out using Student’s *t* test and *p*<0.05 reported when significant. Values represent the average of at least three independent experiments.

## Results

### EBV-encoded dUTPase Induced gene Changes in hDCs

Gene expression patterns provide insight into complex biological networks in which viruses and host cells interact. Dendritic cells are the most potent antigen presenting cells of the immune system and are crucial for the initiation of T-cell responses to viral pathogens. Previous studies performed in our laboratories demonstrated that EBV-encoded dUTPase, an early protein produced during lytic replication of EBV, triggers a signaling cascade through TLR2 that results in the activation of NF-κB and increased secretion of pro-inflammatory cytokines [12]. In addition, the results from our depletion studies [10] indicated that hDCs might be a likely target of the EBV-encoded dUTPase. To better understand the biological effects of the EBV-encoded dUTPase on gene modulation in cells that play a central role in innate and adaptive immune responses, microarray gene expression profiling studies were performed on untreated or EBV-encoded dUTPase treated hDCs using the human genome U133 Plus 2.0 GeneChip, as described in Materials and Methods. The results of this study identified 894 differentially expressed genes between control and EBV-encoded dUTPase treated samples for each normalized data set using the criteria fold change >2 and *p*<0.05. As shown in Table 1, the main pathways affected by EBV-encoded dUTPase in hDCs include cytokine/chemokine and receptor interaction, Toll-like receptor signaling, cell cycle/apoptosis/proliferation pathways, exosome formation and oncogenesis.

**Table 1:** Major Pathways/genes modulated by EBV-encoded dUTPase in human dendritic cells*.

Gene Pathway	Gene Symbol	Gene Name	Fold-change	*P-*Value
**Cytokine/Chemokine Receptor Interaction**				
	IL−1A	Interleukin 1 alpha	108.89	0.010273
	IL−1B	Interleukin 1 beta	6.14	0.000142
	IL−6	Interleukin 6	96.75	0.003221
	IL−10	Interleukin 10	8.6	0.004363
	IL−12B	Interleukin 12p40	9.63	0.00372
	IL−23A	Interleukin 23, alpha subunit p19	5.9	0.004581
	IL−15	Interleukin 15	2.99	0.006863
	IL−15RA	Interleukin 15 receptor alpha	3.71	0.006091
	TNF	Tumor necrosis factor	3.65	0.00107
	TNFSF9	TNF (ligand) superfamily, member 9	9.74	0.001251
	TNFSF10	Trail/TNF (ligand) superfamily, member 10	10.53	0.000616
	TNFSF15	Trail/TNF (ligand) superfamily, member 15	11.76	0.000867
	CCL1	Chemokine (C-C motif) ligand 1	14.39	0.001006
	CCL3	Chemokine ligand 3	16.43	0.000012
	CCL4	Chemokine ligand 4	49.84	0.000441
	CCL8	Chemokine ligand 8	23.28	0.000657
	CCL20	Chemokine ligand 20	335.29	0.007071
	CXCL10	Chemokine (C-×-C motif) ligand 10	140.2	0.002354
	CXCL11	Chemokine ligand 11	62.2	0.004203
	CXCL9	Chemokine ligand 9	7.83	0.005534
	RANTES	Chemokine ligand 5	24.99	0.00677
	INHIBA	Inhibin, beta A	30.75	0.000015
	IFNb1	Interferon 1 beta	11.15	0.003902
	PTX3	pentraxin-related gene	11.38	0.00417
	IL4I1	interleukin 4 induced 1/FIG	2.43	0.000936
	SLAMF1	Signaling lymphocytic activation molecule family member 1	9.77	0.00212
	LTA	lymphotoxin alpha	39.49	0.009009
	CSF1	Colony stimulating factor 1	3.68	0.000484
**Exosome Formation**				
	EXOSC1	Exosome component 1	(−2.8)	0.028162
	EXOSC3	Exosome component 3	3.62	0.015085
	EXOSC6	Exosome component 6	3.7	0.001354
				
**Interferon Inducible Genes**				
	GBP1	Guanylate binding protein 1	20.39	0.000654
	GBP4	Guanylate binding protein 4	15.69	0.00899
	GBP5	Guanylate binding protein 5	31.36	0.00744
	IFI44	Interferon-induced protein 44	6.12	0.005596
	IFI44L	IFN-induced protein 44–like	5.34	0.005429
	IFIH1	IFN-induced protein with helicase C domain 1	6.27	0.004855
	IFIT1	IFN-induced protein with tetratricopeptide repeats 1	20.39	0.000112
	IFIT2	IFN-induced protein with tetratricopeptide repeats 2	64.24	0.034743
	IFIT3	IFN-induced protein with tetratricopeptide repeats 3	34.24	0.001316
	IFIT5	IFN-induced protein with tetratricopeptide repeats 5	10.72	0.000841
	ISG15	Ubiquitin-like modifier induced by interferon	4.96	0.00013
	ISG20	Interferon-stimulated exonuclease gene, 20 KDa	8.55	0.004842
	EBI3	Epstein-Barr virus induced gene 3	10.01	0.002119
	EPSTI1	Epithelial stromal interaction 1	12.92	0.005116
	HERC5	hect domain and RLD 5	6.34	0.000313
	USP18	Ubiquitin-specific peptidase 18	19.4	0.002673
	MX1	Myxovirus resistance 1	4.83	0.000191
	MX2	Myxovirus resistance 2	6.66	0.000236
	OAS1	2′–5′-oligoadenylate synthetase 1	4.03	0.021463
	OAS2	2′–5′-oligoadenylate synthetase 2	7	0.01182
	OAS3	2′–5′-oligoadenylate synthetase 3	16.85	0.014238
	OASL	2′–5′-oligoadenylate synthetase-like	16.19	0.000585
	TRIM2	Tripartite motif-containing 2	3.35	0.001511
	TRIM8	Tripartite motif-containing 8	3.16	0.000164
	TRIM15	Tripartite motif-containing 15	3.05	0.002416
	TRIM21	Tripartite motif-containing 21	2.46	0.004176
	TRIM25	Tripartite motif-containing 25	2.36	0.010354
	TRIM56	Tripartite motif-containing 56	3.01	0.0338
	PML	Promyelocitic leukemia	5.35	0.005078
				
				
**Oncogenesis**				
	BIC	B-cell integration cluster transcript, miR-155 precursor	11.68	0.002143
	MiR223	micro RNA-223 transcript variant 1 mRNA	(−3.21)	0.017805
	OSM	Oncostatin M	4.91	0.005468
	TCF7L2	Transcription factor 7–like 2	3.06	0.005617
	TNC	Tenascin C	23.14	0.007739
	TNFAIP6	TNFα-induced protein 6	15.99	0.001685
	EREG	Epiregulin	15.35	0.005536
	WNT5A	Wingless-type MMTV integration site family, member 5A	72.72	0.002838
	FRAT2	frequently rearranged in advanced T-cell lymphomas 2	(−4.04)	0.000178
	MALAT1	Metastasis associated lung adenocarcinoma transcript 1	4.52	0.008457
	TDGF1	Teratocarcinoma-derived growth factor 1	4.56	0.004075
				
**Cell Cycle/Proliferation/Apoptosis**	BCL6	B-cell CLL/lymphoma 6 (zinc finger protein 51)	(−2.47)	0.000239
	BCL2L1	BCL2-like 1 (Bcl-XL)	4.13	0.003551
	BCL9L	B-cell CLL/lymphoma like 9	3.74	0.000155
	BCOR	BCL6 co-repressor	4.11	0.001152
	CD40	Cluster of differentiation 40	3.38	0.000206
	CDK6	Cyclin-dependent kinase 6	4.22	0.009025
	EMR2	EFG-like module containing mucin-like, hormone receptor-like 2	3.57	0.000034
	FGF9	Fibroblast growth factor ligand 9	3.88	0.032989
	FGF18	Fibroblast growth factor ligand 18	3.77	0.006855
	FOXO1	Forkhead box O1	2.37	0.002695
	G0S2	G0/G1 switch 2	16.83	0.000036
	PDGFRL	Platelet-derived growth factor receptor-like	35.46	0.003063
	XAF1	XIAP associated factor-1	8.05	0.001122
	PDCD1LG2	Programmed cell death 1 ligand 2	3.41	0.030372
	GAS5	Growth arrest-specific 5	(−2.34)	0.000561
	HDAC9	Histone deacetylase 9	(−4.89)	0.031884

* hDC cells were treated with purified EBV-encoded dUTPase or left untreated for 4 h and microarray gene expression profiling performed using the human genome U133 Plus 2.0 genechip as described in Materials and Methods. MBEI normalization was performed using the dChip analysis tool. Differentially expressed genes between control and treated samples were identified for each normalized data set using the criteria fold change >2 and *p*<0.05 (un-paired t-test on the comparison between the expression values of control and treated samples).

Interestingly, several interferon inducible genes involved in anti-viral immune responses including the GTPase-encoding MX1, 2′, 3′ oligoadenylate synthetase 3 (OAS3), OASL, the ubiquitin-like protein modifier ISG15, 3′, 5′ exonuclease ISG20, promyelocytic leukemia (PML), the guanylate binding-proteins GBP1, GBP4 and GBP5 as well as the interferon responsive genes IFI44, IFI44L, IFIH1, IFIT1, IFIT2, IFIT3 and IFIT5 were also up-regulated by EBV-encoded dUTPase (Table 1).

**Figure 1:**
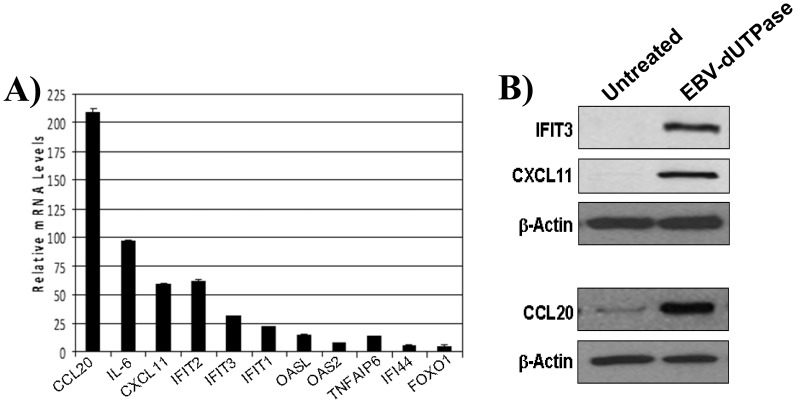
Validation of select target genes up-regulated in microarray studies. **A)** RNA from untreated or EBV-encoded dUTPase treated hDCs for 4 h was examined by qRT-PCR for the expression of CCL20, IL-6, CXCL11, IFIT1, IFIT2, IFIT3, OASL, OAS2, TNFAIP6, IFI44 and FOXO1 using specific TaqMan gene expression assays (ABI). Samples were normalized to GAPDH and expressed as the mRNA expression levels relative to untreated control. **B)** Western blot analysis of CCL20, CXCL11 and IFIT3 using whole cell lysates from untreated or EBV-encoded dUTPase-treated and protein specific Abs (1∶200 dilution).

A representative group of EBV-encoded dUTPase induced genes from the microarray profiling study was validated by qRT-PCR and western blot analysis. As shown in Figure 1A, EBV-encoded dUTPase induced the expression of CCL20, IL-6, CXCL11, IFIT1, IFIT2, IFIT3, OASL, OAS2, TNFAIP6, IFI44 and FOXO1 in hDCs following a 4 h treatment relative to untreated cells as determined by qRT-PCR using specific TaqMan gene expression assays (ABI) (Figure 1A). Samples were normalized to GAPDH and expressed as the mRNA expression levels relative to untreated control. Furthermore, western blot analysis of CCL20, CXCL11, IFIT3 (Figure 1B) using whole cell lysates from untreated or EBV-encoded dUTPase-treated and protein specific Abs further confirmed the results obtained by microarray gene profiling studies. All together, these data provide a modular view of unique hDC responses to EBV-encoded dUTPase and suggests mechanisms by which EBV-encoded dUTPase may modulate immune responses.

### EBV-encoded dUTPase Induces the Secretion of T_H_1 and T_H_17 Cytokines in Human DCs

**Figure 2:**
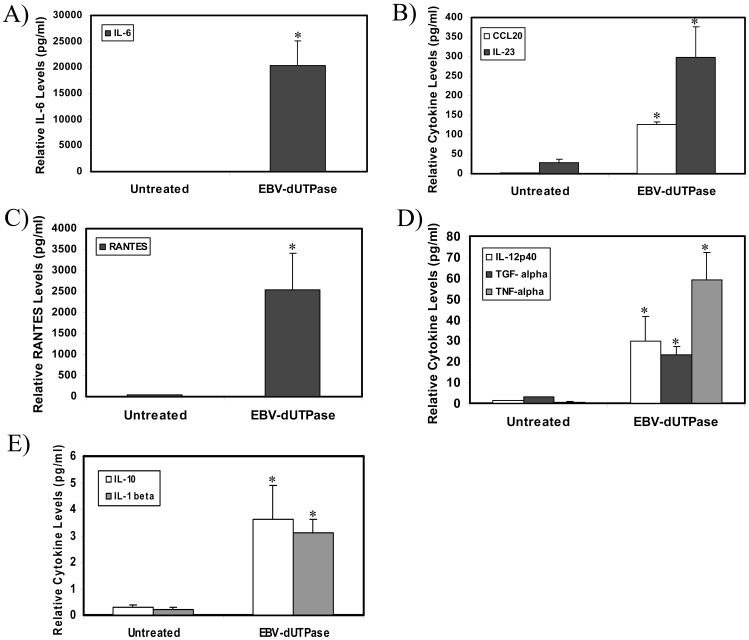
EBV-encoded dUTPase induces the secretion of cytokines and chemokine in human DCs. EBV-encoded dUTPase (10 µg/ml) differentially induce the secretion of T_H_1, T_H_17 cytokines/chemokines in hDCs following a 24 h treatment, as determined by proteome array. (A) IL-6; (B) CCL20 and IL-23; (C) RANTES; (D) IL12p40, TGF-α and TNF-α; (E) IL-10 and IL-1β. Cytokine/chemokine concentrations represent the average ± SD (picograms per milliliter) from two independent experiments (n of 4). *p<0.05.

We have previously demonstrated that the EBV-encoded dUTPase induced the production of pro-inflammatory cytokines in PBMCs and human monocyte derived macrophages [10], [11]. To further examine the biological properties of EBV-encoded dUTPase protein on the production of pro-inflammatory T_H_1 and T_H_17 cytokines, proteome array studies were performed in hDCs treated with EBV-encoded dUTPase, control human dUTPase proteins or left untreated for 24 h, as described in Materials and Methods. As shown in Figure 2, treatment of primary hDCs with EBV-encoded dUTPase recombinant protein resulted in a statistically significant increase in the production of cytokines TNF-α, IL-23, IL12p40, IL-1β, IL-6, IL-10 and TGF-α in comparison to untreated hDCs or hDCs treated with the human nuclear dUTPase control protein (data not shown). While IL-17 levels were slightly elevated in the supernatant of EBV-encoded dUTPase treated hDCs, they were not significant (data not shown). In addition, EBV-encoded dUTPase strongly induced the production of CCL20 and RANTES (*p*<0.05). These results suggest that the EBV-dUTPase has the potential to modulate T_H_17 cell responses.

### EBV-encoded dUTPase is Released from Chemically Induced Raji Cells in Exosomes

**Figure 3:**
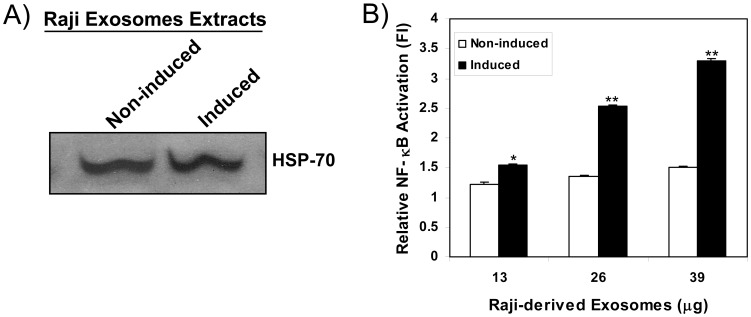
EBV-encoded dUTPase released in exosomes induces NF-κB activation through TLR2. A) Western blot analysis of exosomal marker HSP-70 in extracts from non-induced and induced Raji cells-derived exosomes. B) TLR2-HEK293 expressing cells were transiently transfected with NF-κB reporter gene and pRL-TK transfection control plasmids. After 24–36 h, cells were exposed for 8 h to equal amounts of exosome extracts (13–39 µg) from induced or non-induced Raji cells and luciferase reporter activity measured, as we have described [12]. Data was normalized for transfection efficiency and reporter activity expressed as the mean relative stimulation ± SD of an n = 4. *p<0.05, **p<0.01.

To determine whether EBV-encoded dUTPase could be secreted from EBV-infected cells in exosomes, Raji cells, an EBV-genome positive Burkitt’s lymphoma nonproducing cell line [20], were employed. Raji cells were induced with TPA-sodium butyrate for 72 h and the culture supernatants were either concentrated or used for exosome purification, as described in Materials and Methods. Exosomal extracts from non-induced and induced Raji cells were routinely analyzed for the presence of the exosomal marker HSP-70 by western blot (Figure 3A) prior to being used in the experiments. Culture supernatants and exosomes obtained from non-induced Raji cells served as controls. As shown in Table 2, there was a 2.1-fold (*p*<0.05) and 4.8-fold (*p*<0.01) increase in dUTPase activity in culture supernatants concentrates and exosomes obtained from induced Raji cells, respectively, when compared to non-induced controls. Furthermore, these data demonstrate that there is greater than a 700-fold enrichment of dUTPase activity in exosomes obtained from induced Raji cells when compared to culture supernatants from these cells (total dUTPase activity in concentrated supernatant 0.114 unit/ml vs 8.19 unit/ml in exosomes). Since chemical induction of latent EBV in Raji cells causes abortive-lytic replication, there is no cell lysis, this further supports the data presented in this study and the premise that the EBV-encoded dUTPase is secreted in exosomes. Furthermore, since a previous study had reported that the nuclear isoform of the human dUTPase is secreted in exosomes of B-cells [21], neutralization experiments were performed to determine whether the dUTPase activity detected in the culture supernatant concentrate and exosomes from chemically induced Raji cells was specifically the EBV-encoded dUTPase. The results of this study showed that the dUTPase activity in the culture supernatant concentrate and exosomes from chemically induced Raji cells was inhibited 71% and 63%, respectively, by human serum from a patient with EBV genome positive diffuse large B-cell lymphoma, which we have demonstrated contained specific neutralizing antibodies against the EBV-encoded dUTPase (unpublished data). Conversely, there was less than 11% and 2% inhibition of the dUTPase activity in concentrated culture supernatants and exosomes derived from non-induced Raji cells, respectively (Table 2).

**Table 2:** EBV-encoded dUTPase activity in culture supernatants and exosomes from Raji cells.^a^

Samples	Treatments	dUTPase Activity (Units/ml)	
		No Antibody	Plus Antibody
Culture Supernatants			
	Non-induced	0.018±0.001	0.016±0.001
	Induced	0.038±0.003*	0.011±0.001
Exosomes			
	Non-induced	1.70±0.07	1.67±0.021
	Induced	8.19±0.06**	3.00±0.010

a Raji cells were induced with TPA and sodium butyrate or left untreated as described in Materials and Methods. Following.

a 72 h induction, culture supernatants were either concentrated or used for exosomes purification and the EBV-encoded dUTPase activity determined as described in Materials and Methods.

* *p*<0.05;

** *p*<0.01.

We next investigated whether the levels of EBV-encoded dUTPase protein present in the exosomes of chemically induced Raji cells were sufficient to induce TLR2-mediated activation of NF-κB. As shown in Figure 3B, exposure of TLR2-HEK293 cells to exosomal extracts from induced Raji cells resulted in a statistically significant (*p*<0.05) low level but dose-dependent activation of the NF-κB reporter gene when compared to non-treated controls. Conversely, no activation of the NF-κB reporter gene was observed in HEK293 lacking TLR2 in response to exosomal extracts derived from induced Raji cells (data not shown), which suggests that NF-κB activation is TLR2 dependent.

### The EBV-encoded dUTPase Present in Exosomes Induces the Secretion of Cytokines from hDCs and PBMCs

We next investigated whether the EBV-encoded dUTPase levels present in exosomes were sufficient to induce the secretion of cytokines in hDCs and PBMCs. To address this possibility, cytokine analyses were performed by ELISA using culture supernatants of hDCs treated with either intact exosomes or exosomal extracts from non-induced or chemically induced Raji cells or left untreated for 24 h, as described in Materials and Methods. EBV-encoded dUTPase (0.1 µg/ml or 10 µg/ml) and Pam3csk4 (0.1 µg/ml; a ligand for TLR2) were used as controls. As shown in Table 3, treatment of primary hDCs with either intact or lysed exosomes obtained from chemically induced Raji cells resulted in a statistically significant (*p*<0.01) increase in the production of cytokines IL-10, IL-12p70, IL-1ß, IL-6 and TNF-α in hDCs. Conversely, with the exception of IL-10, there was no significant increase in the production of IL-12p70, IL-1ß or IL-6 by either intact or exosomal extracts from non-induced Raji cells. There was, however, a statistically significant increase (*p*<0.05) in IL-10 induction by the exosomal extract obtained from non-induced Raji cells. More importantly, with the exception of IL-8 and TNF-α the level of cytokine IL-6, IL-1β, IL-12p70 and IL-10 induced by intact exosomes and the exosomal extracts were similar to that observed in hDCs treated with recombinant EBV-encoded dUTPase (0.1 µg/ml; 10 µg/ml).

**Table 3:** Cytokine profile induced by exosomes in hDCs.

Treatments^a^	IL-10 (Pg/ml)	IL12p70 (Pg/ml)	IL-1ß (Pg/ml)	IL-6 (Pg/ml)	IL-8 (Pg/ml)	TNF-α(Pg/ml)
Untreated	0.64±0.61	1.83±0.84	1.75±0.21	1.79±1.05	304.22±1.31	13.58±1
Intact Exosomes Non-induced	1.33±0.23	1.57±0.80	1.77±0.06	1.51±0.56	289.10±1.18	12.48±0.45
Intact Exosomes Induced	9.34±1.48**	37.93±1.95**	14.24±1.09**	43.46±2.90**	4431.60±0.69**	1211±2.34**
Exosome Extract Non-induced	1.71±0.24*	4.26±1.90	1.79±0.66	5.25±2.01*	934.56±1.06**	20.08±4.52*
Exosome Extract Induced	7.22±1.27**	47.57±13.03*	15.43±1.16**	44.45±7.13**	4227.46±12.60**	1113±3.65**
EBV-dUTPase (0.1 µg/ml)	8.58±0.58**	10.25±3.57*	3.54±0.33**	40.93±3.67**	1542.18±1.67**	35.35±6.51*
EBV-dUTPase (10 µg/ml)	73.94±5.20**	49.16±16.3**	7.49±0.78**	208.4±16.9**	11860.4±5.3**	230.82±3.11**
Pam3csk4 (0.1 µg/ml)	9.99±0.80**	9.22±2.38**	3.21±0.23*	22.91±5.51**	2181.36±234.56**	49.64±7.49**

a hDCs were treated with equal amounts of intact or lysed exosomes from non-induced or chemically induced Raji cells, EBV-encoded dUTPase (0.1; 10 µg/ml), Pam3csk4 (0.1 µg/ml) or left untreated, as described in Materials and Methods. After 24 h, culture supernatants were collected and analyzed for cytokines levels using the MSD multi-array/multi-spot human cytokine tissue culture kit. Data represents means ± SD of an n of 4.

* *p*<0.05,

** *p*<0.01.

Similar results were obtained in a parallel study with PBMCs treated with either intact exosomes or exosomal extracts (Table 4). There was no significant production of any of the cytokines examined by exosomes or exosomal extracts from non-induced Raji cells. Conversely, there was a significant induction (*p*<0.05) of IL-10, IL-12p70, IL-1ß, IL-8 and TNF-α, by intact exosomes and the exosomal extracts from chemically induced Raji cells. These results demonstrate that the EBV-dUTPase secreted in exosomes has the potential to modulate hDCs and PBMCs immune responses.

**Table 4:** Cytokine profile induced by exosomes in PBMCs.

Treatments^a^	IFN-γ (Pg/ml)	IL-10 (Pg/ml)	IL12p70 (Pg/ml)	IL-1ß (Pg/ml)	IL-6 (Pg/ml)	IL-8 (Pg/ml)	TNF-α(Pg/ml)
Untreated	3.75±2.84	0.82±0.67	26.46±5.84	0.56±0.30	29.95±10.93	3910.50±133	58.49±23.45
Intact ExosomesUninduced	2.82±0.68	0.96±0.13	16.89±1.53	0.35±0.08	24.82±8.34	4005.35±1173	47.33±5.38
Intact ExosomesInduced	42.31±7.01**	5.30±0.31**	32.98±0.86	1.46±0.10**	38.79±5.61	>4500.51±52**	391.93±34.15**
Exosome ExtractUninduced	3.24±1.45	0.92±0.13	16.63±1.46	0.43±0.07	20.60±1.94	3827.28±194	43.28±6.47
Exosome Extract Induced	28.40±0.35**	3.46±0.13*	38.19±4.80**	1.67±0.06	42.93±4.09	>4520.60±43**	170.81±9.77**
EBV-dUTPase (0.1 µg/ml)	9.82±0.73**	2.48±0.27**	28.29±2.70	1.10±0.07*	58.17±3.61*	>4278.25±66**	123.90±22.53**
EBV-dUTPase (10 µg/ml)	215±16.55**	157±20.6**	301.77±17**	30±2.36**	2499±162**	>45029±665**	8998.64±375**
Pam3csk4 (0.1 µg/ml)	89.99±19**	29.51±7.3**	350.5±56.2**	10.1±1.21**	611.7±131**	>40797±492**	2441±338.17**

a PBMCs were treated with equal amounts of intact or lysed exosomes from non-induced or chemically induced Raji cells, EBV-encoded dUTPase (0.1 or 10 µg/ml), Pam3csk4 (0.1 µg/ml) or left untreated, as described in Materials and Methods. After 24 h, culture supernatants were collected and analyzed for cytokines levels using the MSD multi-array, multi-spot human cytokine tissue culture kit. Data represents means ± SD of an n of 4.

* *p*<0.05,

** *p*<0.01.

### TNF-α Secretion in hDCs and PBMCs by EBV-encoded dUTPase in Exosomes is Mediated by TLR2

As shown above, EBV-encoded dUTPase secreted in exosomes from chemically induced Raji cells stimulates the secretion of pro-inflammatory cytokines in hDCs and PBMCs. To determine whether this process is TLR2 dependent, blocking experiments were performed. hDCS and PBMCs were incubated with anti-TLR2 or isotype control (IgG2a, IgG1) antibodies for 1 h, followed by treatment with intact exosomes, exosomal extracts from non-induced or chemically induced Raji cells or left untreated for 24 h, as described in Materials and Methods. Pam3csk4 (0.1 µg/ml; a ligand for TLR2) was used as a control. After 24 h, culture supernatants from control and treated samples were collected and analyzed for TNF-α levels by ELISA. We chose to measure TNF-α as a representative marker of EBV-encoded dUTPase mediated induction of this group of cytokines. As can be seen by the data presented in Table 5, treatment of hDCs and PBMCS with anti-TLR2 antibody resulted in a statistically significant (*p*<0.01) decrease in the production of TNF-α from hDCs and PBMCs treated with either the exosomal extract or intact exosomes obtained from induced Raji cells. However, pre-incubation of cells with the isotype control antibody did not inhibit exosomes (intact or extract)-mediated stimulation of TNF-α production in hDCs or PBMCs.

**Table 5:** TNF-α production by EBV-encoded dUTPase-containing exosomes in hDCs and PBMCs is TLR2-mediated.

Treatments^a^	hDCs_TNF-α(pg/ml)	PBMCs_TNF-α(pg/ml)
Untreated	13.58±1	58.49±23.45
Intact Exosomes Induced (IEI)	1210.80±2.34	391.93±34.15
IEI+TLR2 Ab	525.91±39.90**	184.67±1.61*
IEI+Isotype Ctl Ab	1045.59±23.12	408.20±13.55
Exosome Extract Induced (EEI)	1112.97±3.65	170.81±9.77
EEI+TLR2 Ab	739.66±22.64**	83.84±40.18**
EEI+Isotype Ctl Ab	1211.29±39.49	123.74±4.82
Pam3csk4 (0.1 µg/ml)	49.64±7.49	2441.05±338.17
Pam3csk4+ TLR2 Ab	14.97±0.18**	1451.74±209.50**
Pam3csk4+Isotype Ctl Ab	43.97±3.46	2343.19±316.14

a hDCs and PBMCs were pre-incubated with anti-TLR2 or isotype control Abs (10 µg/ml) for 1 h and subsequently treated with equal amounts of either intact or lysed exosomes from chemically induced Raji cells, Pam3csk4 (0.1 µg/ml) or left untreated, as described in Materials and Methods. After 24 h, culture supernatants were collected and analyzed for TNF-α levels using the MSD human cytokine tissue culture kit. Data represents means ± SD of an n of 4.

* *p*<0.05,

** *p*<0.01.

The results from these experiments demonstrate that the EBV-encoded dUTPase is released in exosomes from EBV-infected cells undergoing lytic or abortive/lytic replication of EBV and can induce the secretion of pro-inflammatory cytokines in neighboring immune cells through TLR2.

## Discussion

Primary infection of EBV, which occurs in the oropharynx, primarily in the tonsils, results in the establishment of a latent infection in memory B-cells in a significant proportion of the adult population [22]. Periodic reactivation results in productive replication of EBV in plasma cells as well as epithelial cells in the tonsils, which is important for transmission of the virus [22], [23]. hDCs play an important role in pathogen recognition, antigen presentation and modulation of both innate and adaptive immune responses and various subtypes of hDCs are reported to be found in tonsils [24]. In the case of EBV, plasmacytoid DCs [25] and a novel subset of langerin positive immature hDCs [26] have been reported to have potential roles in controlling EBV infections. However, there is essentially nothing known regarding the ability of various EBV-encoded proteins, which are expressed during lytic or abortive-lytic replication of EBV, to modulate DC function. We have previously reported that the EBV-encoded dUTPase activated NF-κB in human monocyte-derived macrophages through TLR2, which resulted in the increased secretion of various pro-inflammatory cytokines [12]. Because of the potential role that hDCs may have in controlling EBV infections, we next determined the biological properties of EBV-encoded dUTPase on gene expression in hDCs and the modulation of the innate and adaptive immune responses.

Recent studies have demonstrated that several EBV-encoded early proteins (BGLF5- alkaline deoxyribonuclease, BNLF2a and BILF1-viral G protein coupled receptor) act as immune evasion molecules by altering MHC-class 1 antigen presentation [27]. The alkaline deoxyribonuclease has also been reported to downregulate the expression of TLR9, which has been implicated in sensing EBV by primary monocytes, plasmacytoid DCs [28], and B-cells [29]. Somewhat surprising is that there have not been any studies, to the best of our knowledge, to determine whether any of the early EBV-encoded proteins may modulate DC function. Type 1 interferons play a major role in establishing an antiviral state by inducing the expression of many genes involved in innate immunity. Studies to elucidate the mechanisms by which viruses are recognized by the innate immune system have focused primarily on viral nucleic acids, since it was assumed that viral encoded proteins lacked conserved motifs that would activate pathogen recognition receptors (PRR) [30]–[32]. These studies have shown that viral nucleic acids induce Type 1 interferon production through TLR7 and TLR9, only in plasmacytoid DCs [28]–[30]. Recently, Barbalat et al [33] demonstrated that inactivated vaccinia virus and mouse cytomegalovirus (MCMV) stimulated the production of Type 1 interferons through a TLR2-dependent pathway in Ly6C^hi^ inflammatory monocytes. Since TLR2 has been reported to recognize several DNA viruses, including members of the herpesvirus family [34]–[36], it suggests that these viruses must contain a protein(s), which is part of the virion, that can target the innate immune system [33]. In the present study, we demonstrate, using microarray gene expression analyses (Table 1), that the EBV-encoded dUTPase up-regulates interferon ß (11.15-fold) as well as five effector pathways of the IFN-mediated antiviral response: the MX1 GTPase pathway, specifically the IFN induced proteins MX1, GBP1, GBP4 and GBP5; the OAS pathway; the ISG20 pathway, specifically members of the tripartite-motif-containing proteins PML, TRIM 19 as well as TRIM 2, 8, 21, 25 and 56; the ISG15 ubiquitin-like protein modifier pathway, which includes HERC5 and USB18 proteins and the IFIT pathway [37]–[39]. Furthermore, microarray analyses demonstrated that the EBV-encoded dUTPase increased the expression of the interferon-inducible T-cell attracting chemokine CXCL-11 by 62-fold in hDCs. Interestingly, these are novel findings mediated by an early protein expressed during lytic or abortive-lytic replication of EBV and suggest that the EBV-encoded dUTPase could prime hDCs to mount an antiviral immune response.

Proteome array analysis of supernatants from untreated or EBV-encoded dUTPase treated hDCs demonstrated that the EBV-encoded dUTPase strongly induced the secretion of pro-inflammatory T_H_1/T_H_17 cytokines including, IL-6, TNF-α, IL-23 and IL-12p40 as well as the chemokines CCL20, IL-8 and RANTES. These chemokines are important in the trafficking of lymphocytes and neutrophils. Interestingly, increased secretion of IL-8, RANTES and CCL20 are known to be up-regulated in EBV associated malignancies [40]. CCL20 is the major chemoattractant for immature DCs, effector/memory T-cells and B-cells through its receptor CCR6. The CCL20-CCR6 axis has been suggested to play a critical role in the initiation of immune responses, especially in the skin and mucosal surfaces [41]. The findings in this study and our previously published work [12] suggest that engagement/stimulation of TLR2 and subsequent activation of NF-κB may lead to increased CCL20 expression, which is consistent with previous studies demonstrating that the expression of CCL20 is mediated by NF-κB [42]. While the role that CCL20 has in the natural EBV-host relationship/interaction remains to be determined, one possibility is that CCL20 enhances the transmission of EBV from B-cells/plasma cells to epithelial cells by promoting the trafficking of B-cells/plasma cells infected with EBV to a microenvironment containing a cellular milieu that is favorable to allowing lytic replication of the virus. Furthermore, these results suggest that CCL20 either alone or in combination with IL-6 may contribute to the proliferation of EBV growth transformed B-cells.

Exosomes are membrane nanovesicles of variable composition that are secreted from multiple cell types into the extracellular space where they have been implicated in several biological processes, including immune surveillance, and are emerging as a potent mechanism of intercellular communication [21], [43]–[50]. Furthermore, numerous studies suggest that various pathogens modulate the immune response through the increase production of exosomes, which activate various signaling cascades through TLRs, including TLR2 [51]–[55]. Several studies have demonstrated that macromolecules encoded by EBV are secreted in exosomes from EBV-transformed lymphoblastoid B cells (LBC) and nasopharyngeal carcinoma cells (NPC) [56]–[59]. Most importantly, these studies revealed that the EBV-encoded macromolecules, which include gp350 [59], latent membrane protein 1 (LMP1) [56], [57] and miRNA [58], modulate cells in the microenvironment. While human and EBV-encoded dUTPases lack consensus secretory signal domains, several studies have reported that the nuclear isoform of the human dUTPase is released from stressed cells [60], [61] and it was recently shown that the human dUTPase is secreted in exosomes from B-cells [21]. Interestingly, the human adenovirus type 9 E4-ORF1 protein, which encodes for an ancestral dUTPase [62], is also targeted to membrane vesicles [63]. A critical and novel finding in our study is the demonstration that the EBV-encoded dUTPase protein is being secreted from chemically induced Raji cells in exosomes. This is supported by at least a 700-fold enrichment of dUTPase activity in exosomal fractions when compared to concentrated culture supernatants from chemically induced Raji cells and a 4.8-fold increase (p<0.01) in dUTPase activity in exosomes derived from chemically induced Raji cells relative to non-induced cells. Perhaps more importantly, these data not only demonstrate that the EBV-encoded dUTPase is being released from EBV-infected cells in exosomes, but that the EBV-encoded dUTPase containing exosomes interact with hDCs through TLR2 to enhance the production of various cytokines at levels that are similar to those observed following treatment of hDCs with purified EBV-encoded dUTPase protein.

**Figure 4:**
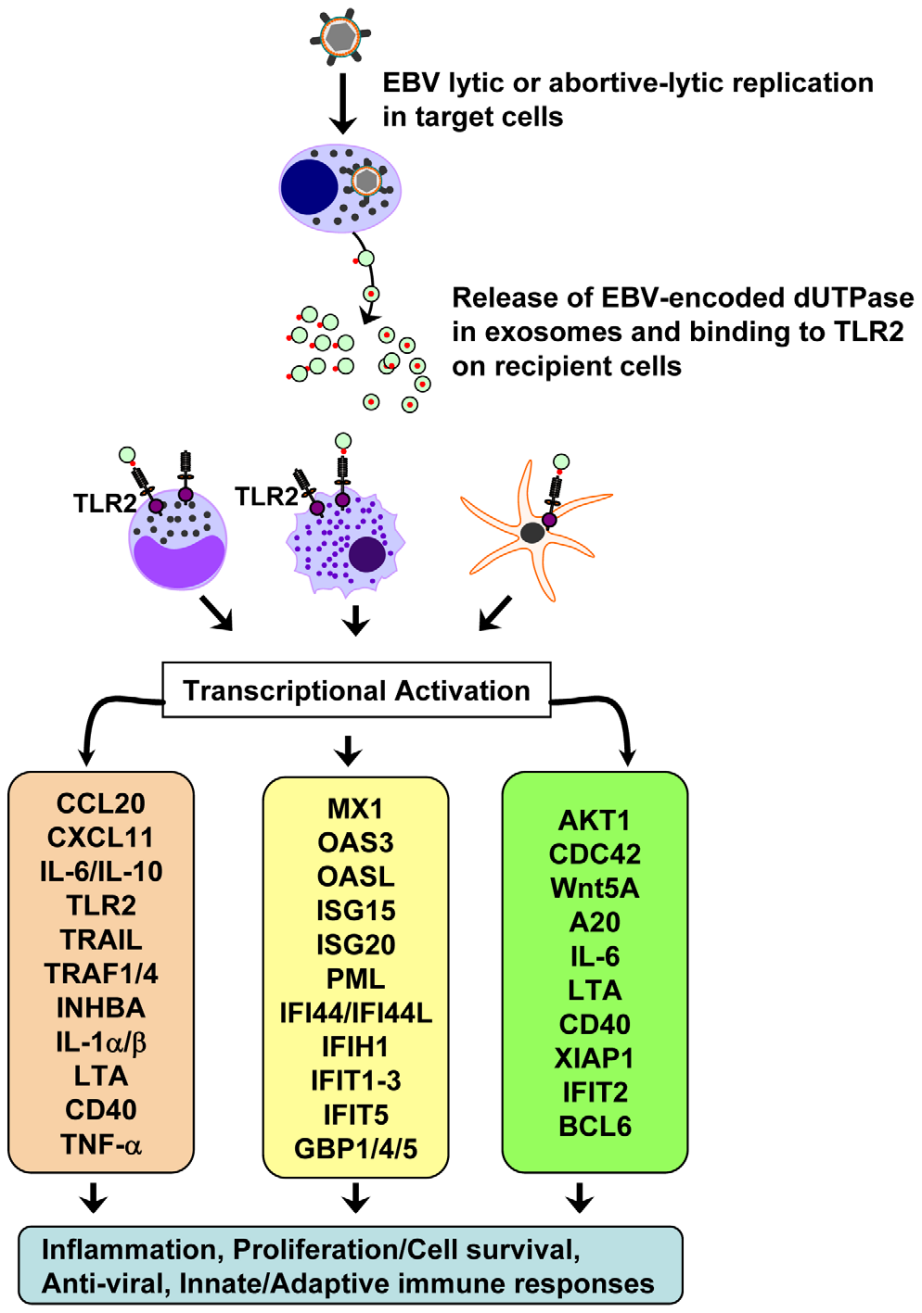
Schematic diagram depicting the novel functions of EBV-encoded dUTPase in innate/adaptive immunity, antiviral response and growth stimulation of B-cells latently infected with EBV. EBV lytic or abortive-lytic replication in target cells results in the expression of the early gene product EBV-encoded dUTPase and subsequent release (free or in exosomes). Binding of EBV-encoded dUTPase to TLR2 in neighboring cells (hDCs, PBMCs) leads to NF-κB activation, followed by induction/secretion of pro-inflammatory cytokines and the up-regulation of genes involved in inflammation, cell survival/proliferation and antiviral response processes. IL-6 acts as a growth factor for B-cells and induces their proliferation.

While EBV is considered to be a B lymphotrophic virus, it infects a variety of cell types including monocytes/macrophages, DCs, neutrophils, natural killer cells and T-cells, but there is no evidence that productive infection occurs in these cells [2]–[4], [64]–[68]. Likewise, it is well established that productive lytic replication occurs in epithelial and plasma cells in vivo. However, the majority of infected cells undergo abortive-lytic replication [2], [3], [64], [65], which does not result in cell lysis but rather apoptosis [22], [69]–[71]. Thus, the primary mechanism for release of EBV encoded macromolecules in cells undergoing abortive-lytic replication may be through exosomes. The data presented in this study demonstrates that the EBV-encoded dUTPase is released in exosomes from B-cells chemically induced to enter an abortive-lytic replicative cycle. The data also demonstrate that the EBV-encoded dUTPase, either free or in exosomes, modulates hDC and PBMC functions through TLR2 by activating a diverse group of signaling pathways that may lead to inflammation, impaired adaptive immunity and cellular proliferation/cell survival (Figure 4). Furthermore, since the EBV-encoded dUTPase can induce chemokines, it has the potential to modulate cell trafficking and attract cells that could become infected by the virus, resulting in its dissemination to other sites within the host. Thus, the EBV-encoded dUTPase may play an important role not only in local but also systemic cell-cell communications by modifying the microenvironment to support/promote the establishment/maintenance of a persistent infection. Although additional studies are necessary to better understand the complex interplay between EBV, EBV-encoded proteins and the host immune system, the data presented in this study suggest that the EBV-encoded dUTPase may play an important role in contributing to the modulation of the EBV life cycle and to the pathology of EBV associated diseases by modulating immune cells’ (hDCs, PBMCs) functions. Interestingly, EBV has been implicated as a possible trigger in several autoimmune diseases including multiple sclerosis [72]–[74], rheumatoid arthritis [75], [76], and systemic lupus erythematosus [77]–[80], as well as in the immune-mediated disease chronic fatigue syndrome [81]. However, the mechanism(s) remains to be elucidated. Our studies open up the possibility of the EBV-encoded dUTPase released in exosomes as a potential mechanism by which this protein could contribute to the altered immune responses that occur in patients with these diseases. Finally, based upon this study and our previous work concerning the EBV-encoded dUTPase [10]–[12], as well as our recent findings with the human endogenous retrovirus encoded dUTPase [18], the data suggest that some virus-encoded dUTPases possess novel immunomodulatory properties that may contribute to the development of various diseases and therefore, they could be potential targets for the development of novel therapeutics.
